# Ectopic Overexpression of *CsECR* From Navel Orange Increases Cuticular Wax Accumulation in Tomato and Enhances Its Tolerance to Drought Stress

**DOI:** 10.3389/fpls.2022.924552

**Published:** 2022-07-05

**Authors:** Dechun Liu, Wenfang Guo, Xinyue Guo, Li Yang, Wei Hu, Liuqing Kuang, Yingjie Huang, Jingheng Xie, Yong Liu

**Affiliations:** Department of Pomology, College of Agronomy, Jiangxi Agricultural University, Nanchang, China

**Keywords:** navel orange, cuticular wax, drought, *CsECR*, transgenic tomato

## Abstract

Drought stress often occurred in citrus to limit its growth, distribution, and fruit quality. Cuticular waxes play an important role in regulating plant tolerance to drought stress. Plant enoyl-CoA reductase (ECR) is involved in the biosynthesis of cuticular waxes and catalyzes the last step of very long-chain fatty acids (VLCFAs) elongation. In this study, a putative *ECR* gene, named *CsECR*, was cloned from “Newhall” navel orange. *CsECR* protein has high identities with other plant ECR proteins and contained a conserved NADP/NAD-binding motif and three conserved functional sites. The highest expression of *CsECR* was observed in leaves, followed by stems, flavedos, ovaries, juice sacs, stigmas, stamens, albedos, and petals. Besides, the expression of *CsECR* was significantly induced by PEG6000 and ABA treatments. Ectopic overexpression of *CsECR* increased the contents of total waxes and aliphatic wax fractions (*n*-fatty acids, unsaturated fatty acids, *n*-alkanes, alkenes, iso-, and anteiso-alkanes) in the leaves and fruits of the transgenic tomato. Furthermore, ectopic overexpression of *CsECR* reduced the cuticle permeability in the leaves and fruits of the transgenic tomato and increased its tolerance to drought stress. Taken together, our results revealed that *CsECR* plays an important role in plant response to drought stresses by regulating cuticular wax biosynthesis.

## Introduction

Cuticular waxes cover plant surfaces and serve as a hydrophobic barrier against biotic and abiotic stresses. In plants, cuticular waxes are mainly composed of very long-chain fatty acids (VLCFAs) and their derivatives with carbon chain lengths ranging from C20 to C34, such as alkanes, primary alcohols, secondary alcohols, aldehydes, esters, and ketones. In addition, cyclic compounds including triterpenoids, alkaloids, and phenylpropanoids are also detected in plant cuticular waxes. The biosynthesis of cuticular waxes started with the *de novo* synthesis of C16 and C18 acyl chains in the plastid of plant epidermal cells. Afterward, the C16 and C18 fatty acids are released from the acyl carrier protein (ACP) and esterified to CoA to produce C16 and C18 fatty acyl CoAs. The C16 and C18 fatty acyl CoAs are further extended to VLCFAs in the endoplasmic reticulum (ER) and generate cuticular wax components by the decarbonylation and acyl reduction pathways (Lee and Suh, 2015a).

The elongation of C16 and C18 fatty acids to VLCFAs in plants includes a series of reaction cycles catalyzed by the multienzyme fatty acid elongase (FAE) complex. Two carbons are added to the acyl chain per cycle. Each cycle consists of four consecutive reactions: biosynthesis of 3-ketoacyl-CoA by condensation of malonyl-CoA with a long chain acyl CoA catalyzed by a 3-ketoacyl-CoA synthase (KCS); reduction of 3-ketoacyl-CoA to 3-hydroxyacyl-CoA catalyzed by a 3-ketoacyl-CoA reductase (KCR); dehydration of 3-hydroxyacyl-CoA to trans-2-enoyl-CoA catalyzed by a 3-hydroxyacyl-CoA dehydratase (HCD); and reduction of trans-2-enoyl-CoA to acyl-CoA catalyzed by an enoyl-CoA reductase (ECR) (Haslam and Kunst, 2013).

To date, a number of genes involved in VLCFA biosynthesis have been reported in plants. For example, the *KCSs* are suggested to harbor fatty acid substrate specificity and determine the chain lengths of the final VLCFA product. A total of 21 *KCS* genes have been identified in the Arabidopsis genome. Among these genes, *KCS1, KCS2/DAISY, KCS6/CUT1/CER6, KCS9, KCS10, KCS13*, and *KCS20* are proved to be involved in cuticular wax synthesis (Millar and Kunst, 1997; Millar et al., 1999; Todd et al., 1999; Lee et al., 2009; Kim et al., 2013). In addition to KCS, the other three enzymes involved in VLCFA biosynthesis have no substrate specificities and take part in the formation of VLCFAs with different carbon chain lengths. There are two *KCR* genes in the Arabidopsis genome, of which only *KCR1* is involved in VLCFA biosynthesis (Beaudoin et al., 2009). However, two *KCR* genes (*GL8A* and *GL8B*) in maize participate in the biosynthesis of VLCFAs (Dietrich et al., 2005). In Arabidopsis, a single *HCD* gene named *PASTICCINO2* (*PAS2*) is involved in VLCFA biosynthesis (Bach et al., 2008).

The first *ECR* gene, named *ScTSC13*, was identified from *Saccharomyces cerevisiae*. The silence of *ScTSC13* decreased the formation of VLCFAs in yeast (Kohlwein et al., 2001). A single *ECR* candidate gene *At3g55360* was identified in the Arabidopsis genome by sequence similarity search with ScTSC13. Further research suggested that the Arabidopsis *cer10* mutant was disrupted in the *At3g55360* gene. Expression of *At3g55360* in the yeast *tsc13* mutant and the Arabidopsis *cer10* mutant revealed that CER10 has functional ECR activity. Furthermore, the biosynthesis of VLCFAs in Arabidopsis *cer10* mutant was interrupted by the *ECR* defect and led to a sharp decrease in cuticular wax load, suggesting that the Arabidopsis *AtECR* gene is involved in VLCFA biosynthesis (Gable et al., 2004; Zheng et al., 2005). Interestingly, the interactions between HCD and other FAE proteins such as KCR and ECR were detected in Arabidopsis, which indicated that HCD is a molecular scaffold for the FAE (Roudier et al., 2010). Further study revealed that the expression of *AtECR* was positively regulated by *AtMYB30* and *AtMYB94*, but negatively regulated by *AtDEWAX* (Raffaele et al., 2008; Go et al., 2014; Lee and Suh, 2015b). In addition to Arabidopsis, the identification of *ECR* homologs was reported in tobacco (Park et al., 2005) and cotton (Song et al., 2009; Mustafa et al., 2017).

Citrus is one of the most important fruit trees in the world. The growth, development, and fruit quality of citrus were severely limited by drought stress. The cuticular waxes limit non-stomatal water loss and play an important role in citrus tolerance to drought stress. To date, the cuticular wax morphology and chemical composition have been studied on many kinds of citrus, such as clementine mandarin (Baker et al., 1975), grapefruit (Nordby and McDonald, 1995), navel orange (Cajuste et al., 2010; Wang et al., 2014; Liu et al., 2015), sweet orange (Ding et al., 2018), satsuma mandarin (Ding et al., 2020), and lemon (Zhou et al., 2021). The molecular mechanism of cuticular wax biosynthesis in citrus has been studied in several reports. For example, the genes involved in the regulation of cuticular wax biosynthesis during fruit development in “Newhall” navel orange were revealed by transcriptomic and metabolomic analyses (Wang et al., 2015). In the past, we reported a navel orange variety, named “Ganqi 3,” originated from a bud mutation of “Newhall” navel orange (*Citrus sinensis* [L.] Osbeck cv. Newhall). Further research showed that many wax-related genes were downregulated on the surfaces of “Ganqi 3” fruits, resulting in a significant decrease in cuticular wax amounts and wax crystal numbers, and finally leading to the glossy surface of “Ganqi 3” fruits (Liu et al., 2012, 2015, 2016). Our recent reports revealed that the expression levels of many wax-related genes changed during cold storage, leading to the alteration of cuticular wax morphology and chemical composition, and finally changed the navel orange tolerances to postharvest decay (Liu et al., 2021). Interestingly, the transcriptomic and metabolomics analyses of another wax-deficient mutant “Gannan NO.1” derived from “Newhall” navel orange revealed that many genes belonging to the VLCFAs elongation and cuticular wax biosynthesis pathways were decreased in “Gannan NO.1” fruits (He et al., 2018). Our group cloned a 3-Ketoacyl-CoA synthase (KCS) gene *CsKCS6* from “Newhall” navel orange. Ectopic overexpression of *CsKCS6* increased the biosynthesis of VLCFAs and enhanced the drought and high salt tolerance of Arabidopsis (Guo et al., 2020). Similarly, ectopic overexpression of *CsMYB30* and *CsMYB96* from *Citrus sinensis* enhanced plant drought resistance by promoting cuticular wax accumulation (Wen et al., 2021; Zhang et al., 2022).

However, previous research focused on screening wax-related genes in citrus by high-throughput sequencing. There are few reports concerned with the function identification of specific wax-related genes in citrus. Under drought stress, plants usually close their stomata to reduce transpiration. However, residual stomatal transpiration even minimal stomatal conductance can interfere with the study of the contribution of plant cuticular waxes to cuticular permeability (Kerstiens, 1996). The astomatous tomato fruits possess a continuous cuticular wax layer relatively impervious to water loss, making it an ideal model for functional characterization of cuticular waxes from plants with defined genetic modifications and therefore revealing the impact of cuticular waxes on plant cuticular permeability and drought tolerance. A series of reports confirmed the important role of the tomato as a suitable model plant to relate cuticular waxes to plant cuticular permeability and drought tolerance. For example, fruits of tomato *lecer6* and *positional sterile* (*ps*) mutants showed a significant increase in cuticular permeability, which was caused by a decline in *n*-alkanes and an increase in triterpenoids (Vogg et al., 2004; Leide et al., 2007, 2011). Overexpression of *SlSHN1* reduced cuticular permeability and enhanced drought tolerance in tomatoes by increasing cuticular wax production (Al-Abdallat et al., 2014). *SlCER1s* and *SlCER3s* tomatoes are involved in the biosynthesis of *n*-alkanes, which are the major cuticular wax components in both tomato leaves and fruits. Overexpression of *SlCER1–1* increases *n*-alkane accumulation in tomato leaves and fruits, therefore enhancing its drought tolerance and fruit storability (Wu et al., 2022). In the present study, a novel cuticular wax biosynthesis gene encoding enoyl-CoA reductase, named *CsECR*, was cloned from “Newhall” navel orange. Moreover, transgenic tomato plants' ectopic overexpressing *CsECR* were produced to determine whether *CsECR* is involved in cuticular wax biosynthesis and plant response to drought stress. The results of this study provide new insights into the molecular regulation of cuticular wax biosynthesis and drought resistance in citrus.

## Materials and Methods

### Plant Materials, Growth Conditions, and Treatments

All citrus tissues were sampled from 8-year-old trees of “Newhall” navel orange (*Citrus sinensis* [L.] Osbeck cv. Newhall) cultivated in an orchard of Xinfeng county, Ganzhou city, Jiangxi province of China. Yong leaves were used for *CsECR* cloning. Mature leaves, stems, flavedos, ovaries, juice sacs, stigmas, stamens, albedos, and petals were used for tissue-specific expression analysis.

For expression analysis after drought and ABA treatments, shoots of “Newhall” navel orange were grafted on trifoliate orange (*Poncirus trifoliata* (L.) Raf.) to produce grafted seedlings. After growing to the height of 30 ± 2 cm in soil culture, the grafted seedlings were transferred to Hoagland's solution in a growth chamber under normal growth conditions (25°C, 16 h light/8 h dark in a day, and 80% relative humidity) for 7 days and then subjected to the Hoagland's solution containing 20% PEG6000 and 100 μM ABA, respectively, for the drought and ABA treatments. For each treatment per biological replicate, the leaves were collected from 15 seedlings at 0, 1, 3, 6, 12, and 24 h. Three biological replicates were performed for each time point of each treatment.

“Micro-Tom” tomato (*Solanum lycopersicum* L. cv. Micro-Tom) was used to produce transgenic plants ectopic overexpressing *CsECR*. For soil culture, seeds of the wild-type “Micro-Tom” tomato (WT) and the transgenic tomato lines were germinated on filter paper in Petri dishes at 25°C in darkness. Germinated seeds were transferred to pots filled with nutritional soil and placed in a growth chamber (25°C, 16 h light/8 h dark in a day, and 80% relative humidity). For *in vitro* culture, tomato plants were grown on a modified MS basal medium at 25°C under a photoperiod of 16 h light/8 h dark. The details of *in vitro* culture were carried out as described in the report by Dan et al. (2006).

### Total RNA Extraction and First-Strand CDNA Synthesis

Total RNA was extracted from different tissues of “Newhall” navel orange, leaves, and fruit peels of “Micro Tom” tomato using the MiniBEST Plant RNA Extraction Kit (TaKaRa, Dalian, China). Trace genomic DNA was removed by DNase I (Promega, Madison, USA). The RNA yield and quality were detected according to our previous report (Liu et al., 2015). The first-strand cDNA was synthesized by PrimeScript™ RT reagent Kit with gDNA Eraser (TaKaRa, Dalian, China) and used for gene clone and expression analysis.

### Cloning and Bioinformatics Analysis of *CsECR*

The homologous cDNA sequence (Cs7g29210) of *CsECR* was obtained from the genomic database of sweet orange (http://citrus.hzau.edu.cn). According to the cDNA sequence of Cs7g29210, a pair of gene-specific primers (sense: 5′ AGAAATAACGAGGGAAGGAT 3′; antisense: 5′ AAAGGCTGCTAGATTACACA 3′) were designed to clone *CsECR* by PCR. The PCR for *CsECR* cloning was performed by 50 μL volume containing 25 μL PrimeSTAR Max Premix (TaKaRa, Dalian, China), 1 μL sense primer, 1 μL antisense primer, 1 μL cDNA, and 22 μL ddH_2_O. The PCR protocol was 5 min at 94°C, 35 cycles of 30 s at 94°C, 30 s at 55 °C, 1.5 min at 72 °C, and finally 10 min at 72°C. The PCR product was cloned into pMD18T vector (Takara, Dalian, China), transformed into *Escherichia coli* DH5α, and sequenced at Shanghai Sangon Biotech as described in our previous report (Liu et al., 2010).

The ExPASy ProtParam tool (http://us.expasy.org/tools/protparam.html) was used to analyze the theoretical isoelectric point and mass values of CsECR protein. The ECR proteins from other plants were obtained by BLAST search in the National Center for Biotechnology Information Server (http://www.ncbi.nlm.nih.gov/). The ClustalW program was used to conduct the multiple sequence alignment. The phylogenetic tree construction was performed by the Minimum-Evolution algorithm of Mega X. The percentage of replicate trees in which the associated taxa clustered together in the bootstrap test (1,000 replicates) are shown next to the branches.

### Expression Analysis of *CsECR* in Navel Orange

Expression profiles of *CsECR* in different tissues of navel orange and after drought and ABA treatments were performed by real-time quantitative PCR (qRT-PCR). The *CsECR*-specific primers (sense: 5′ CGCTTCAGTCATGCAACGTC 3′; antisense: 5′ GGGGTATATCGTGGGTGGTTC 3′) were designed for qRT-PCR analysis. The qRT-PCR was performed on the CFX96 Touch Real-Time PCR Detection cycler (Bio-Rad, Hercules, USA) using the SYBR^®^Premix Ex Taq™ kit (Takara, Dalian, China). The qRT-PCR was performed by 20 μL volume containing 10 μL SYBR^®^Premix Ex Taq, 0.5 μL sense primer, 0.5 μL antisense primer, 1 μL cDNA, and 8 μL ddH_2_O. The qRT-PCR program was 30 s at 95°C for pre-denaturation, 40 cycles of 5 s at 95°C for denaturation, and 30 s at 60°C for annealing. Melting curve analysis of the qRT-PCR products was performed to confirm the amplicon specificity. The relative gene expression levels were normalized relative to the expression of the Citrus β*-actin* gene (sense: 5′ ACTGCTCATTCTCAGC CTATG 3′; antisense: 5′ TGCACCCTGTTCTTCTTACTG 3′) and calculated by the 2^−Δ*ΔCT*^ method.

### Generation of “Micro Tom” Tomato Ectopic Overexpressing *CsECR*

A pair of *CsECR*-specific primers with *Nco* I and *Bgl* II restriction sites (sense: 5′ CGACCATGGAT GAAGGTGACTGTAA 3′; antisense: 5′ TTTAGATCTCTACAGGAATGGGGGC 3′) were used to clone the coding sequence of *CsECR* by RT-PCR. The PCR product was digested by *Nco* I and *Bgl* II and ligated into the pCAMBIA1301 vector to produce the expression vector pCAMBIA1301-*CsECR* under the control of the CaMV35S promoter. The freeze-thaw method was used to introduce this overexpression vector into *Agrobacterium tumefaciens* strain LBA4404. “Micro Tom” tomato ectopic overexpressing *CsECR* was generated by *Agrobacterium*-mediated cotyledon transformation according to the report of Dan et al. (2006). The regeneration of transgenic plants was performed according to the method described by Dan et al. (2006). The positive T_2_ transgenic tomato lines were screened by hygromycin and further confirmed by genomic PCR with a pair of primers (CaMV35S sense primer: 5′ GCCGTAAAGACTGGCGAACA 3′; *CsECR* antisense: 5′ ATTGGCGACGTTGCA TGACT 3′). The genomic PCR was performed by a TransDirect^®^ Plant Tissue PCR Kit (Transgen, Beijing, China). The expression levels of *CsECR* in leaves and fruit peels of the WT and T_2_ transgenic lines were examined by qRT-PCR with the *CsECR*-specific primers as described in the above Section Expression Analysis of *CsECR* in Navel Orange. Tomato *actin* gene (sense: 5′ GAAATAGCATAAGATGGCAGACG 3′; antisense: 5′ ATACCCACCATCACACCA GTAT 3′) was used as the endogenous control to normalize the expression levels between different samples.

### Scanning Electron Microscopy

The cuticular wax morphology on the surfaces of the fully expanded leaves and red ripe fruits in the WT and transgenic tomato lines was observed by SEM. The samples were prepared as described in our previous report (Liu et al., 2015). In brief, fruit peel and leaf disks with a diameter of 0.5 cm were excised from the equatorial zones of the fruits and the leaves. After transferring to aluminum holders, the disks were frozen in liquid N_2_, freeze-dried, and sputter-coated with gold film in a sputter coater (SBC-12, KYKY, Beijing, China). The SEM (Zeiss DSM 962, Carl Zeiss, Oberkochen, Germany) was used to observe the coated disks at ×1,000 and ×5,000 magnification.

### Extraction and Chemical Analysis of Cuticular Waxes

Three biological replicates were carried out for cuticular wax extraction and chemical analysis. A total of 20 fully expanded leaves and 15 red ripe fruits in each line per replicate were used for cuticular wax analysis. The leaves and fruits were immersed three times in chloroform at room temperature for 1 min each time to extract the cuticular waxes. Then, 100 μg of *n*-tetracosane (C24) was added to the cuticular wax solution as an internal standard. The cuticular wax solution was filtered, concentrated by a rotary evaporator at 35°C under reduced pressure, and dried under a gentle stream of nitrogen. Prior to the gas chromatography–mass spectrometry (GC–MS) experiment, the samples were treated with bis-*N*,*N*-(trimethylsilyl)-trifluoroacetamide (BSTFA) in pyridine for 40 min at 70°C to transform the carboxyl and hydroxyl compounds to their corresponding trimethylsilyl ethers and esters. After derivatization, the residual BSTFA was removed under a gentle stream of nitrogen and dissolved in 1 ml of chloroform for GC-MS analysis.

The GC-MS analysis was carried out according to the method of Vogg et al. (2004). In brief, 1 μL of the wax sample was analyzed using Agilent 6890N GC equipped with an HP-5 MS capillary column (30 m × 0.25 mm i.d. × 0.25 μm, Agilent, Palo Alto, CA, USA) and combined with an Agilent 5973N mass spectrometry detector (Palo Alto, CA, USA). Helium was used as the carrier gas with a constant low rate of 2 ml·min^−1^. GC was performed with an on-column injection temperature of 50°C, held for 2 min at 50°C, increased by 40°C·min^−1^ to 200°C, held for 2 min at 200°C, increased by 3°C·min^−1^ to 320 °C, and held for 30 min at 320°C. The MS was performed in positive electron ionization mode at 70 eV, obtaining spectra with an *m/z* range of 50–850. The wax components were identified by matching their electron ionization mass spectra with those from the NIST 14 MS library. The quantitative analysis of the wax components was performed by the same GC system equipped with a name ionization detector (FID). The same chromatographic conditions as above were used to quantify the wax components, but with H_2_ as carrier gas. The wax components were quantified by peak area comparisons with the internal standard. The leaf area was determined using a LI-3100C area meter (Li-Cor, Lincoln, NE, USA). Tomato Analyzer version 3.0 was used to assess the fruit surface area (Brewer et al., 2006). The amounts of wax constituents were expressed per unit of leaf or fruit surface area.

### Measurement of Water Loss Rate and Chlorophyll Leaching Rate

Leaf and fruit non-stomatal water loss rates were investigated as described by Kosma et al. (2009). Briefly, tomato plants were first transferred to dark for 12 h to make sure that stomata were fully closed. Then, 30 fully expanded leaves and 30 red ripe fruits of the same size were detached from tomato plants in each line per replicate. The excised leaves and fruits were placed at room temperature in the dark for 6 h and 10 days, respectively, and weighed on an analytical balance every 1 h for leaves and every 2 days for fruits. Water loss was calculated as a percentage of the initial fresh weight of leaves or fruits at each time point.

For measurement of chlorophyll leaching rate, 20 fully expanded leaves per biological replicate in each line were sampled and soaked in 80% ethanol under a dark environment. After 15, 30, 60, 90, 120, 150, and 180 min of initial immersion, 2 ml of the solution was used to determine the absorbance of the extract at 647 and 664 nm wavelength by a UV-2600 spectrophotometer (Shimadzu, Kyoto, Japan). The leaf chlorophyll concentration was calculated using the following equation: total micromoles chlorophyll = 7.93 (A664) + 19.53 (A647) (Lolle et al., 1998). Chlorophyll leaching rate was expressed as a percentage of the chlorophyll concentration at each time point in total chlorophyll extracted after 24 h. Three biological replicates were used for water loss and chlorophyll leaching analysis.

### Analyses of Phenotype, Ion Leakage, and Survival Rates in WT Plants and Transgenic Lines Under Drought Stress

The 60-day-old seedlings grown in soil were used to compare the difference in phenotype, ion leakage, and survival rates between the WT and two transgenic lines. For drought treatment, the seedlings of the two genotypes were deprived of water for 15 days and then re-watered for 5 days. The control plants of the two genotypes were well-watered during treatment. For measurement of leaf ion leakage, 30 fully expanded leaves in each line per replicate were sampled from each line per replicate after drought treatments. The ion leakage was measured according to the report of Huang et al. (2010). Meanwhile, 30 seedlings in each line per replicate were used to calculate the survival rates under drought stresses. The survival rates were expressed by the percentage of live plants after drought treatment. Three biological replicates were used for the investigation of ion leakage and survival rates.

### Statistical Analysis

All data in this paper are shown as the means ± standard deviations of three biological replicates. Statistical analyses were carried out using Student's *t*-test (for two independent samples) and Duncan's multiple range tests (for one-way ANOVA) in SPSS version 22.

## Results

### Isolation and Sequence Analysis of *CsECR*

Based on the *in silico* cloning and RT-PCR strategy, a novel gene *CsECR* was isolated from the “Newhall” navel orange and deposited in GenBank under accession No. ON146445. The cDNA sequence of *CsECR* is 1,524 bp in length with a 933 bp open reading frame (ORF). The CsECR protein contained 310 amino acids with a predicted molecular mass of 36.11 kDa and an isoelectric point of 9.73.

**Figure 1:**
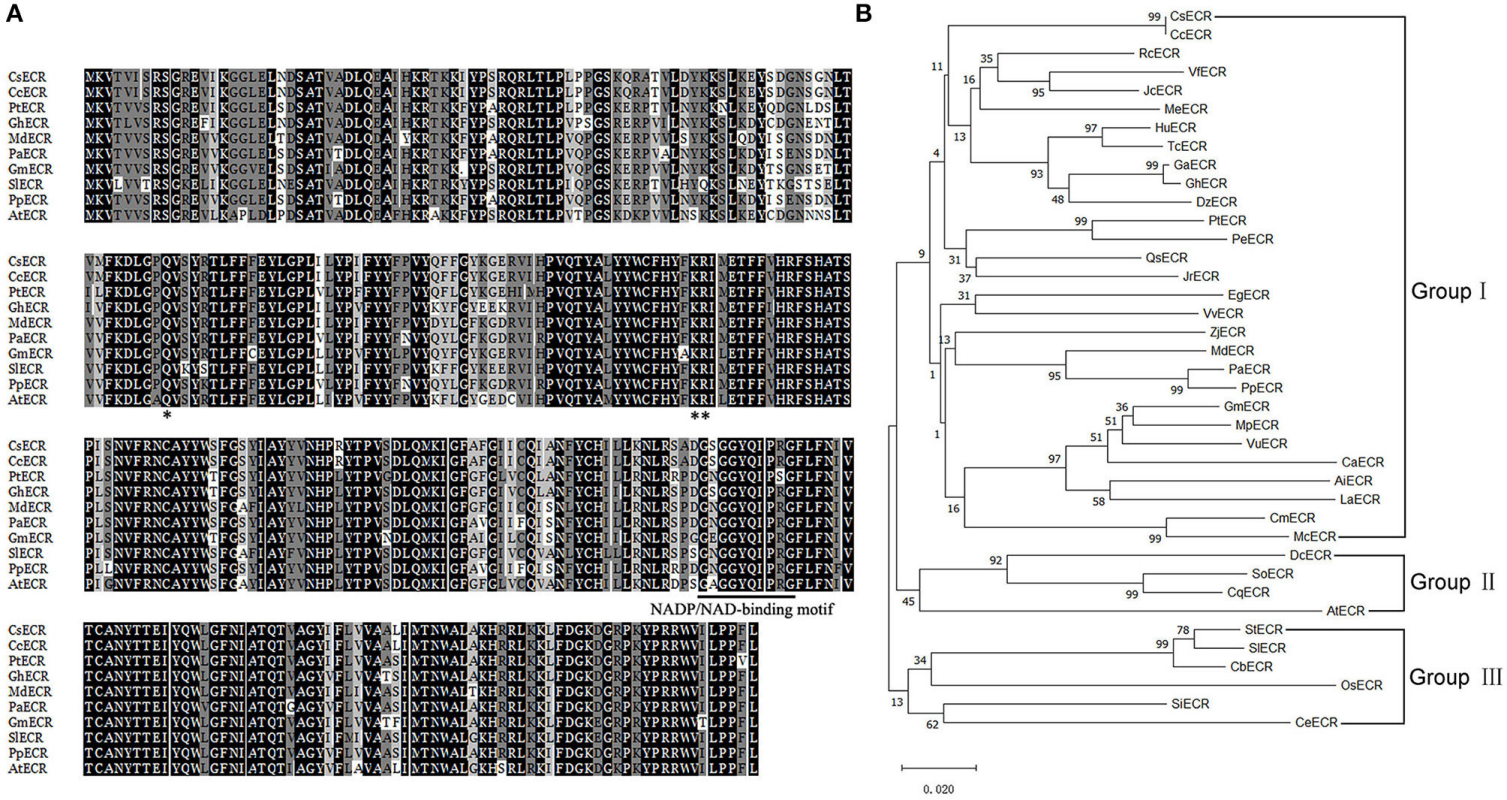
Multiple sequence alignment **(A)** and phylogenetic analysis **(B)** of CsECR with its homologous proteins from different plant species, including *Citrus clementina* CcECR (XP_006435990.1), *Ricinus communis* RcECR (XP_002530850.1), *Vernicia fordii* VfECR (AHI86054.1), *Jatropha curcas* JcECR (XP_012089325.1), *Manihot esculenta* MeECR (XP_021618477.1), *Herrania umbratical* HuECR (XP_021295484.1), *Theobroma cacao* TcECR (XP_007009348.1), *Gossypium arboretum* GaECR (GenBank accession number XP_017620211.1), *Gossypium hirsutum* GhECR (NP_001314306.1), *Durio zibethinus* DzECR (XP_022765174.1), *Populus trichocarpa* PtECR (XP_002311166.1), *Populus euphratica* PeECR (XP_011027192.1), *Quercus sube* QsECR (XP_023916079.1), *Juglans regia* JrECR (XP_018814522.1), *Eucalyptus grandis* EgECR (XP_010067405.1), *Vitis vinifera* VvECR (XP_010658577.1), *Ziziphus jujuba* ZjECR (XP_015876925.1), *Malus domestica* MdECR (NP_001315672.1), *Prunus avium* PaECR (XP_021813755.1), *Prunus persica* PpECR (XP_007218716.1), *Glycine max* GmECR (NP_001276149.2), *Mucuna pruriens* MpECR (RDX83442.1), *Vigna unguiculata* VuECR (XP_027941677.1), *Cicer arietinum* CaECR (NP_001265884.1), *Arachis ipaensis* AiECR (XP_016166715.1), *Lupinus angustifolius* LaECR (XP_019455882.1), *Cucurbita maxima* CmECR (XP_022992762.1), *Momordica charantia* McECR (XP_022134103.1), *Dianthus caryophyllus* DcECR (BAN15750.1), *Spinacia oleracea* SoECR (XP_021855497.1), *Chenopodium quinoa* CqECR (XP_021749534.1), *Arabidopsis thaliana* AtECR (NP_191096.1), *Solanum tuberosum* StECR (XP_006345960.1), *Solanum lycopersicum* SlECR (XP_010321381.1), *Capsicum baccatum* CbECR (PHT49178.1), *Oryza sativa* OsECR (XP_015621959.1), *Sesamum indicum* SiECR (XP_011091470.1), and *Coffea eugenioides* CeECR (XP_027160771.1). The NADP/NAD-binding motif was marked by solid bar. The conserved amino acid residues were marked by asterisk. Construction of the phylogenetic tree was performed with the Neighbor-Joining algorithm of MEGA 4 version.

BLAST analysis revealed that CsECR protein shared high identities with ECR proteins from other plant species, such as *Citrus clementina* CcECR (100%, GenBank accession number XP_006435990.1), *Populus trichocarpa* PtECR (89%, XP_002311166.1), *Gossypium hirsutum* GhECR (88%, NP_001314306.1), *Malus domestica* MdECR (87%, NP_001315672.1), *Prunus avium* PaECR (87%, XP_021813755.1), *Glycine max* GmECR (87%, NP_001276149.2), *Solanum lycopersicum* SlECR (85%, XP_010321381.1), *Prunus persica* PpECR (87%, XP_007218716.1), and *Arabidopsis thaliana* AtECR (83%, NP_191096.1). Multiple sequence alignment showed that CsECR contained a conserved NADP/NAD-binding motif (225-GSGGYQIPRG-234) and three conserved functional sites (Q89, K144, R145) (Figure 1A). To investigate the relationship between CsECR and other plant ECRs, a phylogenetic tree was constructed using the Minimum-Evolution algorithm of the MEGA X version. The investigated ECR proteins could be divided into three groups. CsECR was first classified with *Citrus clementina* CcECR, and then clustered with *Ricinus communis* RcECR, *Vernicia fordii* VfECR, *Jatropha curcas* JcECR, *Manihot esculenta* MeECR, *Herrania umbratical* HuECR, *Theobroma cacao* TcECR, *Gossypium arboretum* GaECR, *Gossypium hirsutum* GhECR, *Durio zibethinus* DzECR, *Populus trichocarpa* PtECR, *Populus euphratica* PeECR, *Quercus sube* QsECR and *Juglans regia* JrECR, *Eucalyptus grandis* EgECR, *Vitis vinifera* VvECR, *Ziziphus jujuba* ZjECR, *Malus domestica* MdECR, *Prunus avium* PaECR, *Prunus persica* PpECR, *Glycine max* GmECR, *Mucuna pruriens* MpECR, *Vigna unguiculata* VuECR, *Cicer arietinum* CaECR, *Arachis ipaensis* AiECR, *Lupinus angustifolius* LaECR, *Cucurbita maxima* CmECR, and *Momordica charantia* McECR, to form the group I. The group II consisted of *Dianthus caryophyllus* DcECR, *Spinacia oleracea* SoECR, *Chenopodium quinoa* CqECR, and *Arabidopsis thaliana* AtECR. The group III included *Solanum tuberosum* StECR, *Solanum lycopersicum* SlECR, *Capsicum baccatum* CbECR, *Oryza sativa* OsECR, *Sesamum indicum* SiECR, and *Coffea eugenioides* CeECR (Figure 1B).

### Expression Analysis of *CsECR* in “Newhall” Navel Orange

**Figure 2:**
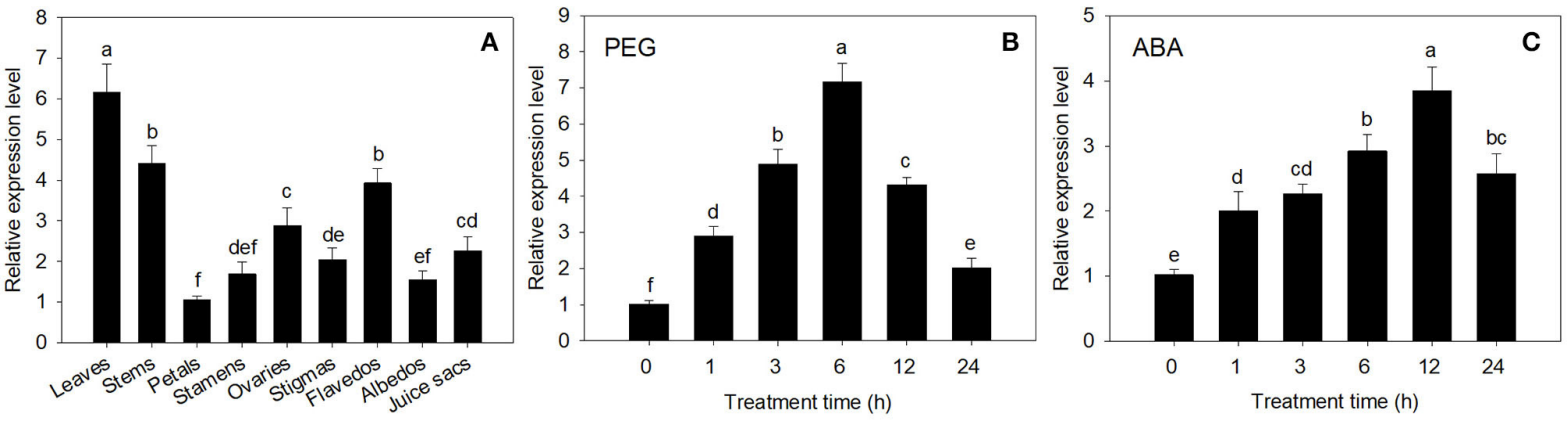
The expression analysis of *CsECR* in “Newhall” navel orange. **(A)** The expression of *CsECR* in different organs. **(B,C)** The expression patterns of *CsECR* in leaves of “Newhall” navel orange after drought (20% PEG 6000) and ABA (100 μM) treatments. The y-axis records the relative gene expression levels as calculated by the 2^−Δ*ΔCT*^ method with β*-actin* as the endogenous reference. Vertical bars represent standard deviations of the means (*n* = 3). Different small letters represent significant differences at *P* < 0.05 according to Duncan's multiple range tests.

The expression of *CsECR* was detected in all of the tested tissues in “Newhall” navel orange. The highest expression was detected in leaves, followed by stems, flavedos, ovaries, juice sacs, stigmas, stamens, albedos, and petals (Figure 2A).

The expression patterns of *CsECR* in leaves of “Newhall” navel orange after PEG6000 and ABA treatments were detected using qRT-PCR. The expression level of *CsECR* was significantly increased at 1 h after treatment with 20% PEG6000 for drought simulation, peaked at 6 h, and then continuously decreased from 6 to 24 h (Figure 2B). Under ABA (100 μM) stress, the *CsECR* expression was significantly increased at 1 h, reached the maximum level at 12 h, and then decreased from 12 to 24 h. These results suggested that the transcript levels of *CsECR* were strongly induced by drought and ABA treatments (Figure 2C).

### Identification of Positive Transgenic Tomato Lines Ectopic Overexpressing *CsECR*

**Figure 3:**
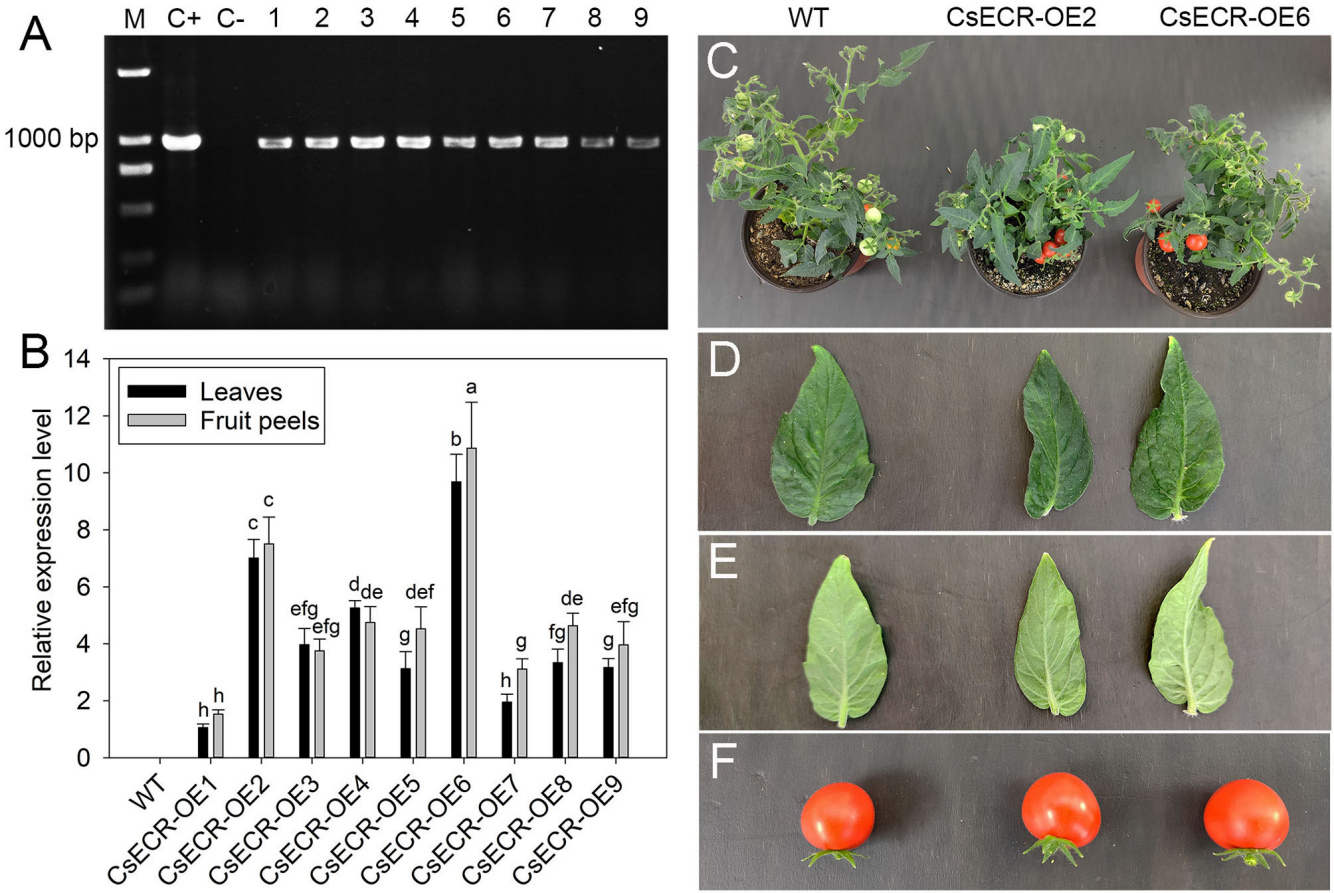
Identification and phenotype analysis of positive transgenic tomato lines. **(A)** Identification of positive transgenic tomato lines by PCR using CaMV35S sense primer and *CsECR* antisense primer. M: standard DNA maker; C+: positive control (plasmid contained recombinant expression vector pCAMBIA1301-CsECR); C–: DNA from the wild-type (WT) tomato leaves. Number 1–9: positive transgenic tomato lines. **(B)** Expression levels of *CsECR* in leaves and fruit peels of the WT and positive transgenic tomato lines (CsECR-OE1 to CsECR-OE9) were investigated by qRT-PCR. **(C)** Phenotype of the WT and two positive transgenic tomato lines. **(D, E)** Phenotype of the adaxial and abaxial sides of leaves, and **(F)** fruits of the WT and two positive transgenic tomato lines (CsECR-OE2 and CsECR-OE6). Vertical bars represent standard deviations of the means (*n* = 3). Different small letters represent significant differences at *P* < 0.05 according to Duncan's multiple range tests.

To analyze the physiological function of *CsECR*, we generated transgenic tomato plants that ectopic overexpressed *CsECR* under the control of CAMV 35S promoter. A total of nine positive T_2_ transgenic tomato lines were confirmed by kanamycin screening and by PCR at the DNA level (Figure 3A). The qRT-PCR results revealed that high transcript levels of *CsECR* were detected in leaves and fruit peels of all positive transgenic tomato lines, but no transcripts could be observed in the WT plants. Two T_2_ transgenic tomato lines (CsECR-OE2 and CsECR-OE6) with the highest expression of *CsECR* in leaves and fruit peels were selected for further research (Figure 3B). The growth behavior of the two transgenic lines was similar to the WT plants under normal growth conditions (Figure 3C). In addition, the leaves and fruits of the two genotypes exhibited similar morphological characteristics under normal growth conditions (Figures 3D–F).

### Ectopic Overexpression of *CsECR* Increases Cuticular Wax Accumulation on the Surfaces of Leaves and Fruits of the Transgenic Tomato Plants

**Figure 4:**
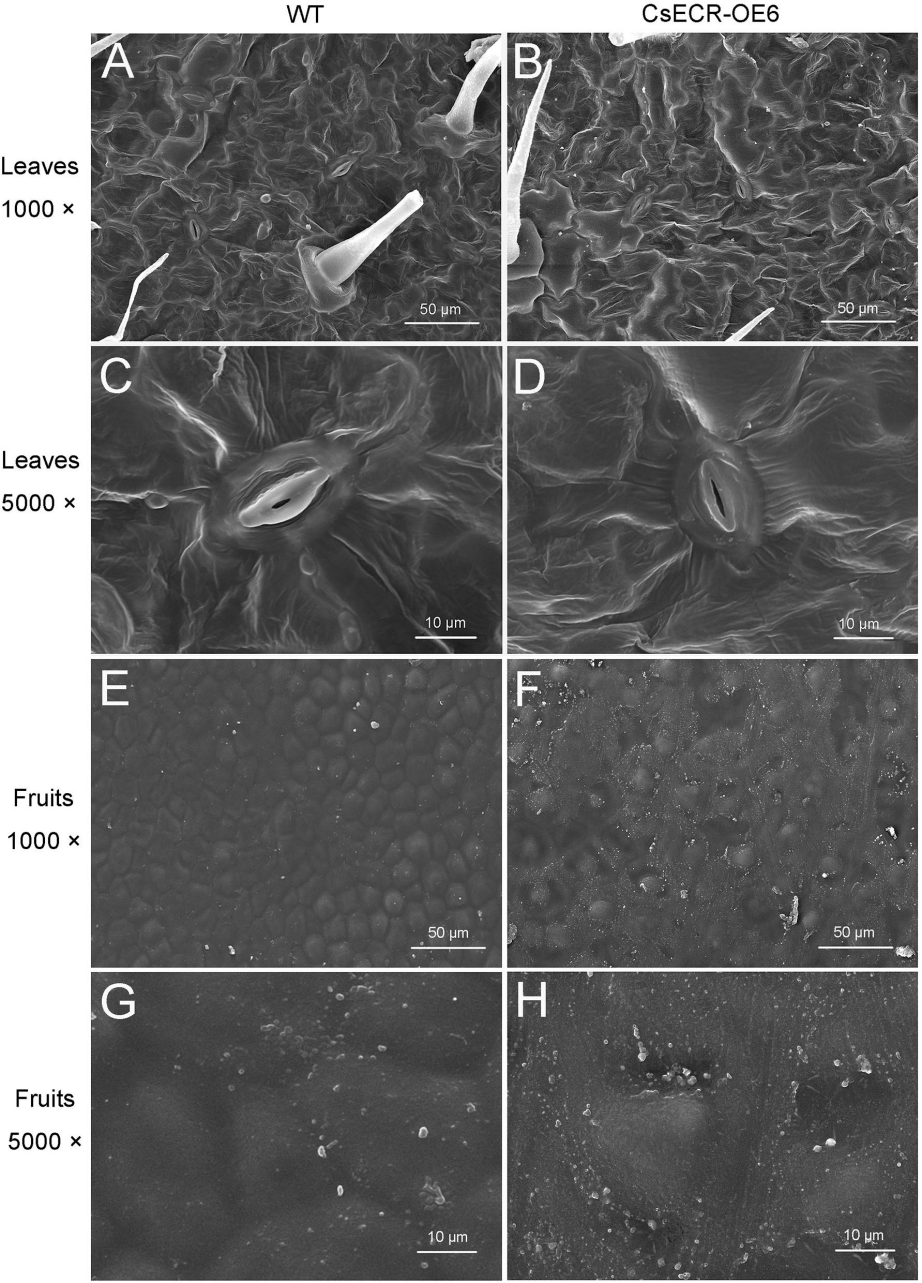
Scanning electron micrographs of cuticular waxes on the surfaces of leaves and fruits in the WT and transgenic tomato (CsECR-OE6). **(A,B)** The abaxial surfaces of leaves in the WT and transgenic tomato line were imaged at ×1,000 magnification. **(C,D)** The abaxial surfaces of leaves in the WT and transgenic tomato line were imaged at ×5,000 magnification. **(E,F)** The surfaces of fruits in the WT and transgenic tomato line were imaged at ×1,000 magnification. **(G,H)** The surfaces of fruits in the WT and transgenic tomato line were imaged at ×5,000 magnification.

SEM results showed that no significant difference in cuticular wax morphology was observed between the abaxial leaf surfaces of the WT and transgenic plants (Figures 4A–D). However, the wax crystal density on the fruit surfaces of the transgenic plants was much higher than that on the WT fruit surfaces (Figures 4E–H).

**Figure 5:**
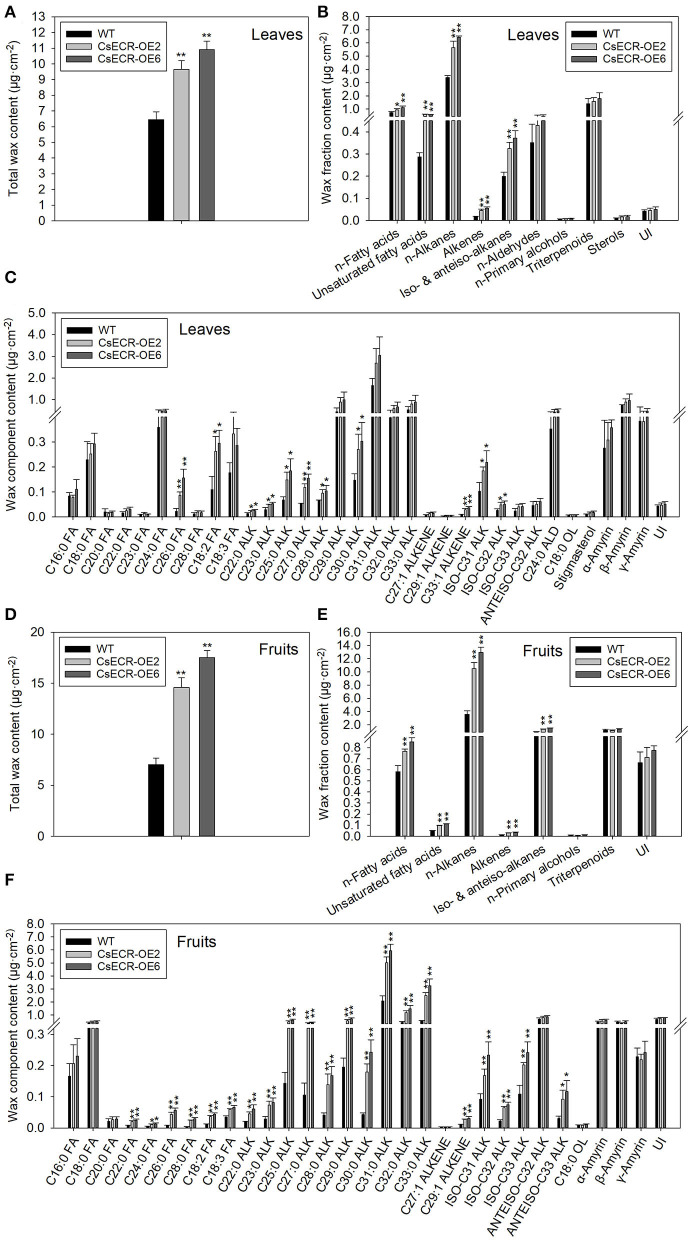
The contents of total waxes, wax fractions, and wax constituents of **(A–C)** leaves and **(D–F)** fruits in the WT and two transgenic tomato lines (CsECR-OE2 and CsECR-OE6). FA, fatty acid; ALK, alkane; ALD, aldehyde; OL, alcohol; UI, unidentified components. Vertical bars represent standard deviations of the means (*n* = 3). Significant differences between the WT and transgenic tomato lines at the *P* <0.05 and *P* <0.01 levels were indicated by single and double asterisks, respectively, according to Student's *t*-test.

**Figure 6:**
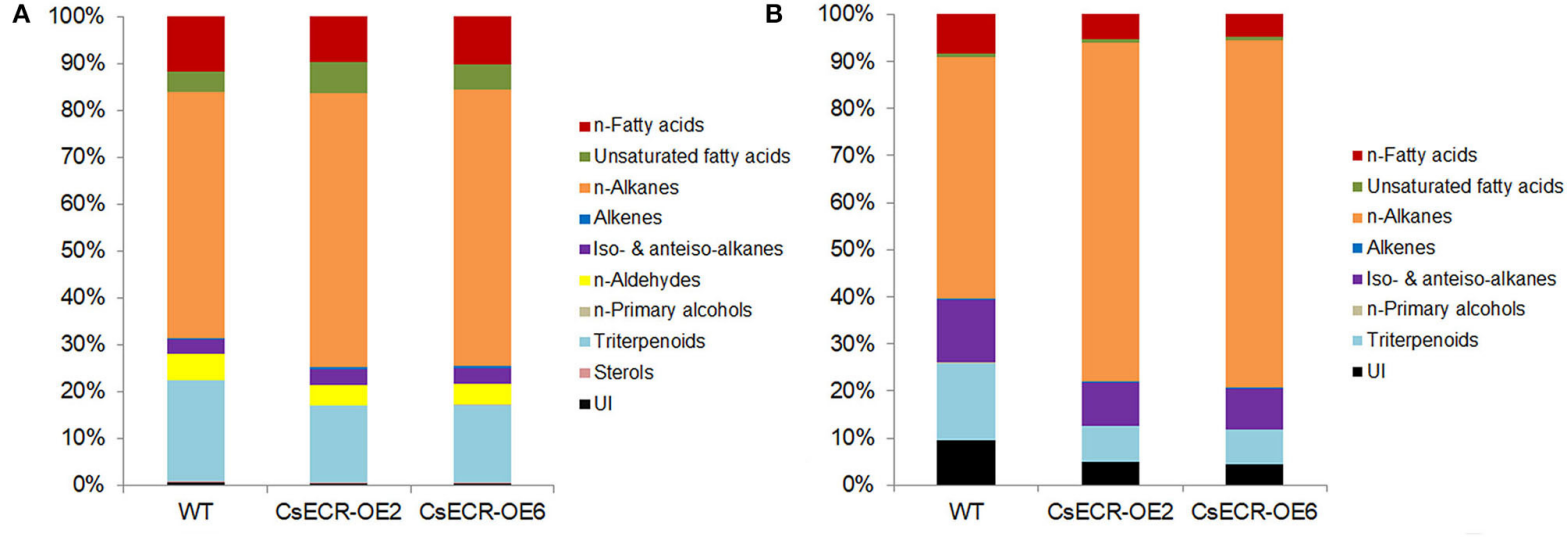
Relative content (%) of each wax fraction in **(A)** leaves and **(B)** fruits of the WT and two transgenic tomato lines (CsECR-OE2 and CsECR-OE6). Values are shown as the means of three biological replicates.

The total wax loads in the leaves of the two transgenic tomato lines were 9.64 and 10.91 μg·cm^−2^, which increased by 49.46 and 69.15%, respectively, in comparison to the WT leaves (6.45 μg·cm^−2^) (Figure 5A). The most abundant cuticular wax fraction in the WT leaves was *n*-alkanes (3.39 μg·cm^−2^, accounting for 52.56% of total wax load), followed by triterpenoids (1.39 μg·cm^−2^, 21.55%), *n*-fatty acids (0.75 μg·cm^−2^, 11.63%), *n*-aldehydes (0.35 μg·cm^−2^, 5.43%), unsaturated fatty acids (0.29 μg·cm^−2^, 4.50%), iso-, and anteiso-alkanes (0.20 μg·cm^−2^, 3.10%), unidentified wax component (0.043 μg·cm^−2^, 0.67%), alkenes (0.018 μg·cm^−2^, 0.28%), sterols (0.0089 μg·cm^−2^, 0.14%), and *n*-primary alcohols (0.0059 μg·cm^−2^, 0.092%). The amounts of *n*-alkanes increased by 66.67% (5.65 μg·cm^−2^) and 89.38% (6.42 μg·cm^−2^), respectively, in the two transgenic lines compared to the WT leaves. Notably, the increases in *n*-alkane amounts accounted for 70.85 and 67.94% increases in total wax loads, respectively, in the two transgenic line leaves. Compared to the WT leaves, the amount of *n*-fatty acids increased by 26.67% (0.95 μg·cm^−2^) and 49.33% (1.12 μg·cm^−2^), respectively, in the two transgenic line leaves. Similarly, the content of unsaturated fatty acids increased by 106.90% (0.60 μg·cm^−2^) and 100.00% (0.58 μg·cm^−2^), the alkenes increased by 155.56% (0.046 μg·cm^−2^) and 211.11% (0.056 μg·cm^−2^), and the iso- and anteiso-alkanes increased by 60.00% (0.32 μg·cm^−2^) and 85.00% (0.37 μg·cm^−2^) in the two transgenic line leaves compared with the WT leaves. However, no significant differences were observed in the amounts of *n*-aldehydes, *n*-primary alcohols, triterpenoids, and unidentified wax components between the leaves of the two genotypes (Figures 5B, 6A). The *n*-alkanes in both genotype leaves were made of C22, C23, C25, C27, and C28 to C33 homologs. The amounts of C22:0, C23:0, C25:0, C27:0, C28:0, and C30:0 alkanes in the two transgenic line leaves were significantly higher than those in the WT leaves. The *n*-fatty acids in the leaves of both genotypes were composed of a series of homologs with even-numbered chain lengths from C16 to C28 and C23. Compared to the WT leaves (0.023 μg·cm^−2^), ectopic overexpression of *CsECR* increased the amounts of C26:0 fatty acid by 278.26% (0.087 μg·cm^−2^) and 595.65% (0.16 μg·cm^−2^), respectively, in the two transgenic line leaves. The unsaturated fatty acids consisted of C18:2 and C18:3 fatty acids in the leaves of both genotypes. The amounts of C18:2 fatty acid in the two transgenic line leaves increased by 136.36% (0.26 μg·cm^−2^) and 172.73% (0.30 μg·cm^−2^), respectively, in comparison to the WT leaves (0.11 μg·cm^−2^). Alkene was composed of C27:1, C29:1, and C33:1 alkene in the leaves of both genotypes. The amounts of C33:1 alkene increased by 218.18% (0.028 μg·cm^−2^) and 320.45% (0.037 μg·cm^−2^) in the two transgenic line leaves compared with the WT leaves (0.0088 μg·cm^−2^). A total of three iso-alkanes (C31–C33) and one anteiso-alkane (C32) were observed in the leaves of both genotypes. The amounts of C31 and C32 iso-alkanes in the two transgenic line leaves were significantly higher than those in the WT leaves. However, no significant changes were observed for the amounts of other wax components (C24:0 aldehyde, C18:0 primary alcohol, stigmasterol, α-amyrin, β-Amyrin, γ-Amyrin, and unidentified wax component) in the two transgenic line leaves (Figure 5C).

Compared to the WT fruits (7.01 μg·cm^−2^), the total wax loads increased by 108.43% (14.59 μg·cm^−2^) and 150.29% (17.52 μg·cm^−2^), respectively, on the surfaces of the two transgenic line fruits (Figure 5D). The most abundant wax fraction in the WT fruits was *n*-alkanes (3.60 μg·cm^−2^, accounting for 51.36% of total wax amounts), followed by triterpenoids (1.15 μg·cm^−2^, 16.41%), iso- and anteiso-alkanes (0.94 μg·cm^−2^, 13.41%), unidentified wax component (0.67 μg·cm^−2^, 9.56%), *n*-fatty acids (0.58 μg·cm^−2^, 8.27%), unsaturated fatty acids (0.048 μg·cm^−2^, 0.68%), alkenes (0.013 μg·cm^−2^, 0.19%), and *n*-primary alcohols (0.0085 μg·cm^−2^, 0.12%). Ectopic overexpression of *CsECR* increased the amounts of *n*-alkanes by 191.94% (10.51 μg·cm^−2^) and 259.17% (12.93 μg·cm^−2^), iso- and anteiso-alkanes by 42.55% (1.34 μg·cm^−2^) and 61.70% (1.52 μg·cm^−2^), *n*-fatty acids by 32.76% (0.77 μg·cm^−2^) and 46.55% (0.85 μg·cm^−2^), unsaturated fatty acids by 100.00% (0.096 μg·cm^−2^) and 129.17% (0.11 μg·cm^−2^), and alkenes by 130.77% (0.030 μg·cm^−2^) and 161.54% (0.034 μg·cm^−2^) in the fruits of two transgenic lines. Notably, 91.16 and 88.77% increases in the total wax loads in the two transgenic line fruit were attributed to the increases of *n*-alkanes (Figures 5E, 6B). A total of 10 *n*-alkane components (C22, C23, C25, and C27–C33) were observed in the fruits of both genotypes. The amounts of all *n*-alkane components in the two transgenic line fruit were significantly higher than those in the WT fruit. The most abundant wax component in the WT fruit was C31 alkane (2.07 μg·cm^−2^, accounting for 29.53% of total wax amounts), which increased by 142.03% (5.01 μg·cm^−2^) and 186.47% (5.93 μg·cm^−2^) in the two transgenic line fruits. Notably, the increases of C31 alkane in the two transgenic line fruit accounted for 38.79 and 36.73% increases in the total wax loads. A total of three iso-alkanes (C31–C33) and two anteiso-alkanes (C32, C33) were observed in the fruit of both genotypes. The amounts of all iso-alkanes and C33 anteiso-alkanes in the transgenic line fruit were significantly higher than those in the WT fruit. The *n*-fatty acids in the fruit of both genotypes consisted of series homologs with even chain lengths ranging from C16 to C28. The amounts of *n*-fatty acids with an even chain length larger than C22 significantly increased in the two transgenic line fruit compared to the WT fruits. Besides, the amounts of two unsaturated fatty acids (C18:2 and C18:3) and C29:1 alkene in the two transgenic line fruits were significantly higher than those in the WT fruits. However, other wax components including C27:1 alkene, C18:0 primary alcohol, α-amyrin, β-Amyrin, γ-Amyrin, and unidentified wax components shared similar amounts between the fruits of the two genotypes (Figure 5F).

### Ectopic Overexpression of *CsECR* Reduces the Leaf and Fruit Cuticle Permeabilities and Enhances the Drought Tolerance of Transgenic Tomato Plants

**Figure 7:**
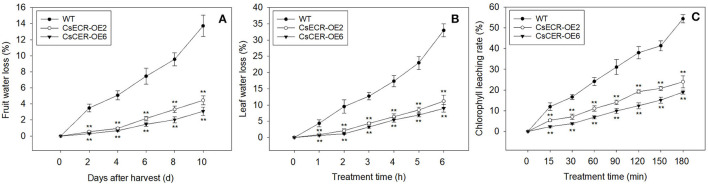
Analyses of cuticular permeability in the fruits and leaves from the WT and two transgenic tomato lines (CsECR-OE2 and CsECR-OE6). **(A)** The water loss of fruits and **(B)** leaves in the WT and two transgenic tomato lines. **(C)** The chlorophyll leaching rates in the leaves of the WT and two transgenic tomato lines. Vertical bars represent standard deviations of the means (*n* = 3). Significant differences between the WT and transgenic tomato lines at the *P* <0.01 levels were indicated by double asterisks according to Student's *t*-test.

Compared to the WT fruits, the fruits of two transgenic lines showed significantly lower water loss rates from 2 to 10 days after harvest (Figure 7A). Similarly, the water loss rates in the leaves of two transgenic lines were also much lower than those in the WT leaves after detachment from plants for 1–6 h (Figure 7B). Moreover, the two transgenic lines possessed much lower leaf chlorophyll leaching rates in comparison to the WT plants after 15–180 min alcohol treatment (Figure 7C). These results indicated that ectopic overexpression of *CsECR* reduces the cuticle permeabilities in the leaves and fruits of transgenic tomato plants.

**Figure 8:**
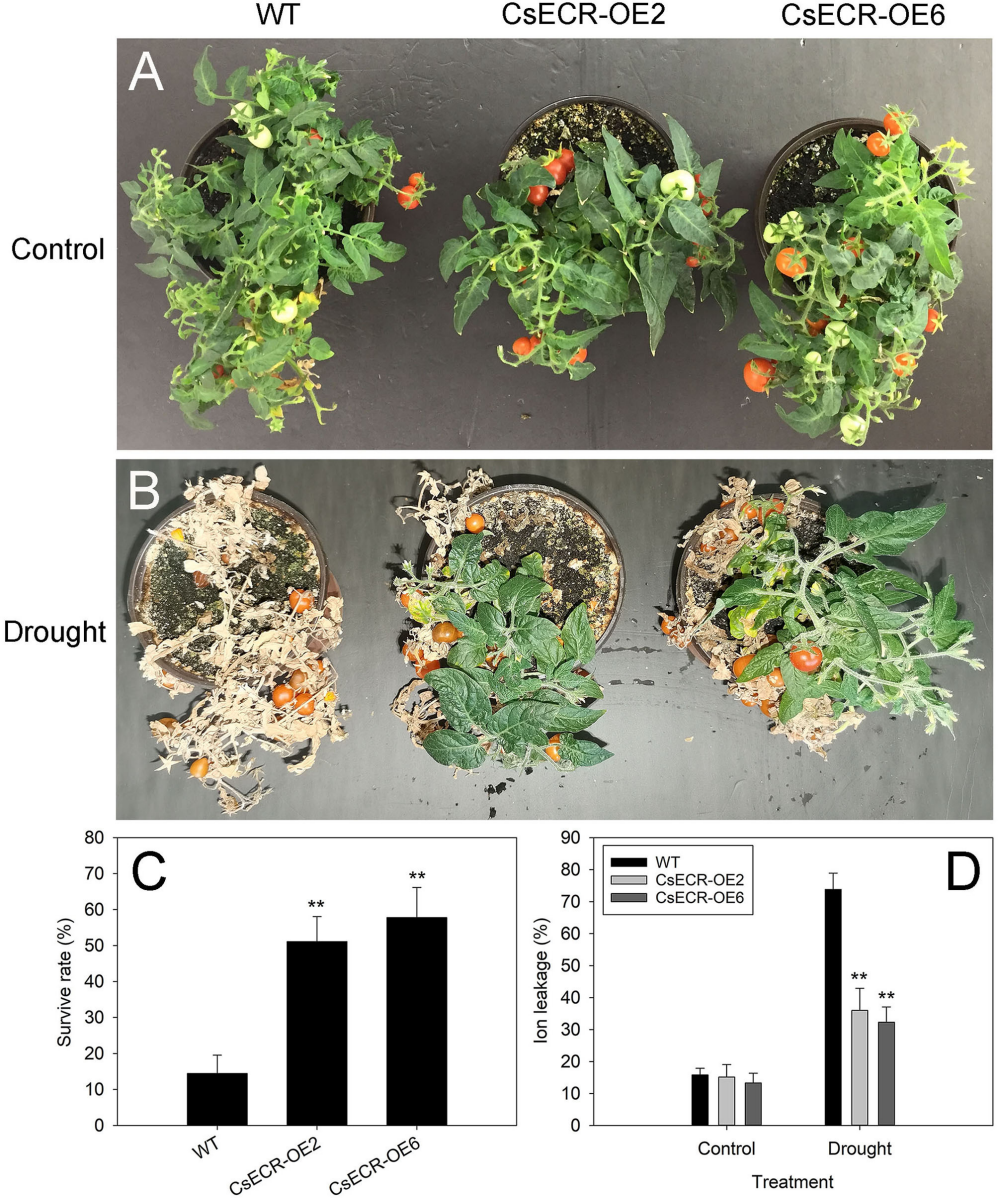
Phenotype **(A,B)**, survival rate **(C)**, and ion leakage **(D)** in the WT and two transgenic tomato lines (CsECR-OE2 and CsECR-OE6) under drought stress (without watering for 15 days before re-watering for 5 days). Vertical bars represent standard deviations of the means (*n* = 3). Significant differences between the WT and transgenic tomato lines at the *P* <0.01 levels were indicated by double asterisks according to Student's *t*-test.

After 15 days of drought treatment and 5 days of recovery, almost all leaves in the WT plants were severely wilted, while the two transgenic lines recovered well with only a few wilting leaves detected at the bottom (Figures 8A,B). The survival rates of the two transgenic lines were much higher than those in the WT plants after drought treatment (Figure 8C). In addition, the ion leakage in the leaves of the two transgenic lines was significantly decreased compared with that of the WT plants after drought treatment (Figure 8D). These results suggested that ectopic overexpression of *CsECR* increased the transgenic plant's resistance to drought stress.

## Discussion

Drought is the main environmental stress that limits citrus growth and reduces its fruit quality and economic value (Rodríguez-Gamir et al., 2010). Our recent report revealed that cuticular waxes play an important role in limiting citrus non-stomatal water loss and regulating the citrus response to drought stress (Liang et al., 2022). However, the underlying molecular mechanism, especially the contribution of specific wax biosynthesis gene to drought tolerance is still unclear in citrus. VLCFAs are precursors and important components of plant cuticular waxes. An FAE system consists of four enzymes that catalyze a series of cyclic reactions to produce VLCFAs in plants. ECR has been shown to catalyze the final step of plant VLCFAs biosynthesis (Haslam and Kunst, 2013). Transcriptome and qRT-PCR analyses in our previous study showed that the expression level of *CsECR* was significantly downregulated in the fruit peels of “Ganqi 3,” a wax-deficient mutant derived from “Newhall” navel orange, suggesting that *CsECR* might be involved in cuticular wax biosynthesis and drought response in citrus (Liu et al., 2016). To prove this hypothesis, *CsECR* was cloned from “Newhall” navel orange and ectopic overexpressed in “Micro-Tom” tomato, a model plant suitable for functional analysis of cuticular waxes due to its astomatous fruit surfaces. CsECR showed high identities with other plant ECR proteins. In addition, all conserved motifs and functional sites of ECR were observed in CsECR. For example, the NADP/NAD-binding motif (225-GSGGYQIPRG-234) characterized in cotton GhECR and other plant ECR (Song et al., 2009), the functional site of K144, R145 in AtECR (Paul et al., 2007) and Q81 (Q89 in CsECR) in *Saccharomyces cerevisiae* (Kohlwein et al., 2001) were also conserved in CsECR (Figure 1A). The phylogenetic analysis showed that CsECR was classified with other plant ECR proteins, especially CcECR, RcECR, VfECR, JcECR, MeECR, HuECR, TcECR, GaECR, GhECR, and DzECR, which were closer to CsECR than other CsECR proteins (Figure 1B). The protein structural features and phylogenetic results suggested that CsECR was a member of the plant ECR family.

Previous reports showed that the expression levels of *TaECR* from wheat (*Triticum aestivum*), *BnECR* from oilseed rape (*Brassica napus*), *MdCER10* (*ECR* homolog) from apple (*Malus domestica*) in leaves and stems were significantly higher than those in other tissues (Ni et al., 2011; Qi et al., 2019; Kong et al., 2020). Similarly, *CsECR* exhibited the highest expression level in leaves, followed by stems and flavedos (Figure 2A), suggesting that it might regulate cuticular wax biosynthesis mainly in these organs. Environmental stresses, such as drought, cold, and UV irradiation, have a great influence on the biosynthesis of plant cuticular waxes (Shepherd and Griffiths, 2006; Xue et al., 2017; Trivedi et al., 2019). It has been reported that many wax biosynthesis genes in plants are induced by drought stress. For example, the expression levels of *AtKCS6* in Arabidopsis, *PpCER1-2* in *Poa pratensis*, and *CsKCS6* in “Newhall” navel orange increased under drought stress (Joubès et al., 2008; Guo et al., 2020; Wang et al., 2021). The *ECR* homolog *MdCER10* from apple showed increased transcript levels after drought and salicylic acid treatments (Qi et al., 2019). Furthermore, the transcript levels of numerous wax biosynthesis genes increased after ABA treatment (Kosma et al., 2009; Seo et al., 2009; Wang et al., 2012). In addition, an Arabidopsis transcription factor MYB96 is proven to regulate cuticular wax biosynthesis by the ABA-mediated signaling pathway (Seo et al., 2011). In agreement with these reports, the expression of *CsECR* was induced by drought and ABA treatments (Figure 2B), suggesting that *CsECR* might be involved in citrus response to drought stress through the ABA-dependent pathway. Plant *ECR* genes play an important role in the biosynthesis of VLCFAs and other wax components. For example, virus-induced gene silencing of *NbECR* in tobacco reduced the amounts of VLCFAs (Park et al., 2005). Silencing of *AtECR* in Arabidopsis and *TaECR* in bread wheat decreased the amounts of total waxes, VLCFAs, and their derivatives such as alkanes, alcohols, aldehydes, and ketone (Zheng et al., 2005; Kong et al., 2020). In accordance with these reports, ectopic overexpression of *CsECR* significantly increased the amounts of total waxes, *n*-fatty acids, unsaturated fatty acids, *n*-alkanes, alkenes, iso-, and anteiso-alkanes in the leaves and fruits of transgenic tomato plants (Figures 5A–F), indicating that *CsECR*, like *AtECR, NbECR*, and *TaECR*, functions as an important gene responsible for the biosynthesis of VLCFAs and other aliphatic wax components. The cuticular wax composition detected in the WT leaves and fruits was similar to previous reports on “Micro Tom” tomato (Vogg et al., 2004; Leide et al., 2007). The *n*-alkanes dominated the culticular wax components in the leaves and fruit of “Micro Tom” tomato (Vogg et al., 2004; Leide et al., 2007). In this study, *n*-alkanes were also the most abundant wax fraction (accounting for >50% of total wax loads) and contributed to the major increases in total wax amounts (contributing to >67% increases in total wax loads in the leaves and fruits of transgenic tomatoes). Surprisingly, the *n*-fatty acids, which were the final product of ECR, increased much less than the *n*-alkanes in both leaves and fruits of the transgenic tomato plants (Figures 5B,E, 6A,B). This result suggested that a larger number of increased *n*-fatty acid components might be converted to *n*-alkanes and other aliphatic wax components in the leaves and fruits of transgenic tomatoes. A previous report revealed that the cuticular wax crystals on the fruit surfaces of tomatoes were mainly composed of aliphatic wax compounds (Vogg et al., 2004). Thus, the significant increases in the amounts of *n*-alkanes and other aliphatic wax components could explain the increases in cuticular wax crystal densities on the fruit surfaces of transgenic tomato plants (Figures 4E–H, 5E,F). To our surprise, the WT and transgenic tomato plants shared similar cuticular wax crystal morphology on the abaxial leaf surfaces, although the total waxes and aliphatic wax components increased significantly in transgenic tomato plant leaves (Figures 4A–D, 5B,C). It should be noted that the increases in total waxes (49.46 and 69.15%) and *n*-alkanes (66.67 and 89.38%) in leaves were much lower than those in fruits of two transgenic tomato lines (increased by 108.43 and 150.29% in total waxes, and 191.94 and 259.17% in *n*-alkanes) (Figures 5A,B,D,E). Thus, not like fruits, the increases of cuticular wax components might not be high enough to self-assemble into the cuticular wax crystals on the transgenic tomato leaf surfaces. Another explanation was that the increased cuticular waxes might cover the leaf surfaces of transgenic tomatoes with a morphology of thin film which could not be observed by SEM.

Previous reports showed that the increases of cuticular waxes could reduce the cuticle permeability and enhance the plant tolerance to drought stress. For example, Arabidopsis and rice plants overexpressing wheat *TaCER1-1A* gene exhibited significant increases of *n*-alkanes, decreases in cuticle permeability, and enhanced tolerance to drought stress (Li et al., 2019). Ectopic overexpression of navel orange *CsKCS6* in Arabidopsis decreased the cuticle permeability and enhanced the transgenic plant tolerance to drought stress by promoting cuticular wax accumulation (Guo et al., 2020). In fact, various wax biosynthesis genes from different plant species have been reported to enhance plant tolerance to drought stress by increasing wax production and reducing the cuticle permeability, such as *AtWXP1* and *AtWXP2* from Arabidopsis, *AhKCS1* from groundnut (*Arachis hypogaea*), *MdSHINE2* and *MdLACS2* from apple (*Malus* × *domestica*), *PpCER1* from *Poa pratensis*, and so on (Zhang et al., 2007, 2019, 2020; Lokesh et al., 2019; Wang et al., 2021). The plant *ECR* genes have been reported to be involved in normal shoot development, cell expansion, membrane biogenesis, and fungal pathogen resistance (Park et al., 2005; Zheng et al., 2005; Kong et al., 2020). However, it is still unknown whether plant *ECR* genes are involved in plant response to drought stress. Our results showed that the increases in cuticular wax contents led to the decreases in cuticle permeability in the leaves and fruits of transgenic tomato plants. Further study revealed that the two transgenic tomato lines were much more tolerant to drought stress than the WT plants, as indicated by their fewer wilting leaves, significantly higher survival rates and much lower ion leakage under drought stress (Figures 8A–D). Our findings proved for the first time that *CsECR*, an *ECR* homologous gene encoding enoyl-CoA reductase in navel orange, has a function of enhancing plant drought tolerance by increasing cuticular wax accumulation. However, further study must be performed to investigate the *CsECR* function in citrus other than in model plants by gene overexpressing and gene silencing techniques. Moreover, the results of this study will prompt us to further investigate the detailed molecular mechanism of *CsECR* regulating the cuticular wax biosynthesis and drought tolerance, including analysis of *CsECR* promotor, identification of the upstream transcription factor, and interacting protein of *CsECR*.

## Conclusion

In summary, a novel gene *CsECR* encoding enoyl-CoA reductase was cloned from “Newhall” navel orange. *CsECR* exhibited differential transcriptional levels in the tested tissues of “Newhall” navel orange, with the highest expression in leaves. The expression of *CsECR* was induced by PEG6000 and ABA. Further study revealed that ectopic overexpression of *CsECR* increased the amounts of *n*-fatty acids, unsaturated fatty acids, *n*-alkanes, alkenes, and iso- and anteiso-alkanes, decreased the cuticle permeability, and enhanced the transgenic tomato tolerance to drought stress. These results suggested that *CsECR* was involved in the biosynthesis of citrus cuticular waxes and plays an important role in regulating plant tolerance to drought stress.

## Data Availability Statement

The original contributions presented in the study are publicly available. This data can be found here: NCBI; ON146445.

## Author Contributions

DL: performed major experimental works, writing the original draft, and acquiring financial funding. WG: performed some physiological experiments and visualization of data. XG: performed statistical analyses. LY: visualization of data, and writing—review and editing. WH: interpreting the data. LK: reviewing of data. YH: visualization of data. JX: writing—review and editing. YL: conception, design of the study, writing—review and editing, and financial funding. All authors reviewed and approved the final manuscript.

## Funding

This study was supported by the National Key Research and Development Program of China (Grant No. 2019YFD1000100), the National Natural Science Foundation of China (Grant Nos. 31701896 and 31860544), the Natural Science Foundation of Jiangxi Province (Grant No. 20202BAB205001), and by the earmarked fund for Jiangxi Agriculture Research System (Grant No. JXARS-07-cultivation post).

## Conflict of Interest

The authors declare that the research was conducted in the absence of any commercial or financial relationships that could be construed as a potential conflict of interest.

## Publisher's Note

All claims expressed in this article are solely those of the authors and do not necessarily represent those of their affiliated organizations, or those of the publisher, the editors and the reviewers. Any product that may be evaluated in this article, or claim that may be made by its manufacturer, is not guaranteed or endorsed by the publisher.
